# Increased Incidence of Vestibular Disorders in Patients With SARS-CoV-2

**DOI:** 10.1097/ONO.0000000000000051

**Published:** 2024-04-02

**Authors:** Lawrance Lee, Evan French, Daniel H. Coelho, Nauman F. Manzoor, on behalf of the N3C consortium., Adam B. Wilcox

**Affiliations:** 1 Department of Otolaryngology – Head and Neck Surgery, School of Medicine, Virginia Commonwealth University, Richmond, Virginia; 2 Wright Center for Clinical and Translational Research, Virginia Commonwealth University, Richmond, Virginia.

**Keywords:** COVID-19, Dizziness, SARS-CoV-2, Vestibular disorder

## Abstract

**Objective:**

Determine the incidence of vestibular disorders in patients with SARS-CoV-2 compared to the control population.

**Study Design:**

Retrospective.

**Setting:**

Clinical data in the National COVID Cohort Collaborative database (N3C).

**Methods:**

Deidentified patient data from the National COVID Cohort Collaborative database (N3C) were queried based on variant peak prevalence (untyped, alpha, delta, omicron 21K, and omicron 23A) from covariants.org to retrospectively analyze the incidence of vestibular disorders in patients with SARS-CoV-2 compared to control population, consisting of patients without documented evidence of COVID infection during the same period.

**Results:**

Patients testing positive for COVID-19 were significantly more likely to have a vestibular disorder compared to the control population. Compared to control patients, the odds ratio of vestibular disorders was significantly elevated in patients with untyped (odds ratio [OR], 2.39; confidence intervals [CI], 2.29–2.50; *P* < 0.001), alpha (OR, 3.63; CI, 3.48–3.78; *P* < 0.001), delta (OR, 3.03; CI, 2.94–3.12; *P* < 0.001), omicron 21K variant (OR, 2.97; CI, 2.90–3.04; *P* < 0.001), and omicron 23A variant (OR, 8.80; CI, 8.35–9.27; *P* < 0.001).

**Conclusions:**

The incidence of vestibular disorders differed between COVID-19 variants and was significantly elevated in COVID-19-positive patients compared to the control population. These findings have implications for patient counseling and further research is needed to discern the long-term effects of these findings.

SARS-CoV-2 is a novel betacoronavirus that causes the clinical syndrome termed COVID-19, which caused a global pandemic leading to 7 million deaths worldwide. Many of the disease’s effects and complications have now been extensively studied and documented in the literature (1–3).

Within the field of otolaryngology, the most common sequelae associated with COVID-19 are chemosensory deficits (ie, smell and taste), with prevalence as high as 47.4% (4). Contributing authors to our current study have also investigated the prevalence of chemosensory deficits associated with COVID-19, demonstrating differences in the rate of chemosensory dysfunction between COVID-19 variants (5).

However, other head and neck symptoms related to COVID-19, particularly of the cochleovestibular system, also necessitate our attention, as up to 58% of patients affected by COVID-19 reported vestibular symptoms and 9% reported auditory symptoms upon initial diagnosis (6). These symptoms have also been found to affect women disproportionately (7). Jeong et al have demonstrated a mechanism of viral entry into the cells of the cochleovestibular end organs via the angiotensin-converting enzyme 2 receptor pathway, providing further insight into the impact it has on hearing loss, tinnitus, and vertigo (8–10).

In this study, we investigated the incidence of vestibular and balance disorders associated with COVID-19 variants and compared it to the general population within the same timeframe using data from a large multi-institutional database.

## MATERIALS AND METHODS

Data from the National Center for Advancing Translational Sciences National COVID Cohort Collaborative (N3C) (11) were utilized for analysis of this study. To compare the impact that variants have on vestibular symptoms, the “peak period” for each variant was determined using covariants.org (12). The “peak period” for each variant (initial/untyped, alpha, delta, omicron 21K, omicron 23A) was defined as the 12 weeks with the highest prevalence of that particular variant in the United States. The peak period date ranges for initial/untyped variant ranged from July 6, 2020 to September 28, 2020, alpha variant from March 22, 2021 to June 14, 2021, delta variant from August 9, 2021 to November 1, 2021, omicron 21K variant from December 27, 2021 to March 21, 2022, and omicron 23A variant from February 3, 2023 to March 28, 2023. The N3C database was queried for patients with positive COVID-19 tests within each variant peak and a vestibular disorder diagnosis occurring within 2 weeks of the test date. Patients with no documented history of COVID-19 were used as the control population for each wave. Because this group represents the general population, each control patient is included in the control population for every wave where their corresponding institution had contributed data at least through the end of the outcomes period. The diagnosis of a vestibular disorder diagnosis was defined by SNOMED code 271789005, which is equivalent to R42 dizziness and giddiness diagnosis code (ICD-10). Relative rates of vestibular disorders were calculated using patients without a recorded COVID diagnosis by querying for unique patients with vestibular disorder diagnosis codes during a 4-week period in the middle of each 12-week variant period. Statistical analysis between groups was performed by odds ratio (OR) calculations (95% confidence interval [CI]) and log odds significance testing using MedCalc (13). This study was conducted under data use request RP-64CD77 and approved by VCU IRB HM20022747. Authorship was determined using ICMJE recommendations.

## RESULTS

Demographic data is displayed in Table 1. The total number of patients with COVID-19 and those with COVID-19 and vestibular disorders by variant are presented in Table 2.

**TABLE 1.** Demographics of patients with COVID-19 and vestibular disorder, by variant

	Initial/untyped	Alpha	Delta	Omicron 21K	Omicron 23A
Age (SD)	51.7 (18.9)	51.9 (17.8)	52.2 (18.7)	53 (19.3)	61.5 (19.3)
Sex (%)					
Female	57.0	57.4	57.4	60.2	58.6
Male	43.0	42.6	42.6	39.8	41.4
Race (%)					
White	54.5	56.0	68.1	62.8	69.9
Black	23.8	26.9	20.4	21.6	15.9
Other/Unknown	21.7	17.1	11.5	15.6	14.2

**TABLE 2.** Incidence of vestibular disorder, by variant period

	Untyped	Alpha	Delta	Omicron 21K	Omicron 23A
Control	10,570,030	10,570,030	9,768,954	9,683,469	8,617,497
Control w/VD	37,480	39,883	35,443	31,269	27,127
COVID+	258,683	170,734	426,736	936,508	56,596
COVID+ w/VD	2182	2314	4656	8928	1530
Odds ratio	2.39^a^	3.63^a^	3.03^a^	2.97^a^	8.80^a^
95% CI	2.29–2.50	3.48–3.78	2.94–3.12	2.90–3.04	8.35–9.27

a *P* < 0.001.

VD indicates vestibular disorder.

### Combined Data

At the time of our analysis, 12,419,287 patients met the inclusion criteria for the time intervals of interest. Of these patients, 1,849,257 patients, or 14.89%, tested positive for COVID-19, and 19,610 of 1,849,257 patients, or 1.06%, tested positive for COVID-19 with our diagnosis of interest. The remaining 10,570,030 patients, or 85.11%, had no documented history of COVID-19 infection.

### Variant Specific Data

There were 10,828,713 patients within the initial/untyped peak period. 37,480 of 10,570,030 patients, or 0.35%, of control patients had a diagnosis of vestibular disorder, compared to 2182 of 258,683, or 0.84%, testing positive for COVID-19 with this diagnosis (OR, 2.39; CI, 2.29–2.50; *P* < 0.001).

A total of 10,740,764 patients were included within the peak period of the alpha variant. 39,883 of 10,570,030 patients, or 0.38%, of control patients had a diagnosis of vestibular disorder, compared to 2314 of 170,734, or 1.36%, testing positive for COVID-19 with this diagnosis (OR, 3.63; CI, 3.48–3.78; *P* < 0.001).

Within the peak period of the delta variant, there were 10,195,690 patients. 35,443 of 9,768,954 patients, or 0.36%, of control patients had a diagnosis of vestibular disorder, compared to 4656 of 426,736, or 1.09%, testing positive for COVID-19 with this diagnosis (OR, 3.03; CI, 2.94–3.12; *P* < 0.001).

A total of 10,619,977 patients were included within the peak period of the omicron 21K variant. 31,269 of 9,683,469 patients, or 0.32%, of control patients had a diagnosis of vestibular disorder, compared to 8928 of 936,508, or 0.95%, testing positive for COVID-19 with this diagnosis (OR, 2.97; CI, 2.90–3.04; *P* < 0.001).

Lastly, 8,674,093 patients were included within the peak period of the omicron 23A variant. 27,127 of 8,617,497 patients, or 0.31%, of control patients had a diagnosis of vestibular disorder, compared to 1530 of 56,596, or 2.70%, testing positive for COVID-19 with this diagnosis (OR, 8.80; CI, 8.35–9.27; *P* < 0.001).

### Intervariant Risk Comparison

As demonstrated previously, patients testing positive for COVID-19 are often 2 to 3 times more likely to develop symptoms of vestibular dysfunction. However, the incidence of vestibular disorders between variants can vary significantly as well. Table 3 illustrates the likelihood of developing vestibular disorders between each variant wave, with the data suggesting that individuals testing positive for the omicron 23A variant are at highest likelihood of vestibular disorder, being 8.80 times greater than the general population, as previously noted, but also being 2.02–3.27 times more likely compared to other variants. On the other hand, patients with the untyped variant appear to be at the lowest risk of developing vestibular symptoms, being 2.39 times more likely than the general population, but at 0.31 the rate compared to individuals affected by the omicron 23A variant.

**TABLE 3.** Intervariant odds ratio comparison of vestibular disorder

Variant	Untyped	Alpha	Delta	Omicron 21K	Omicron 23A
Untyped	–	0.62 (0.58–0.66)	0.77 (0.73–0.81)	0.88 (0.84–0.93)	0.31 (0.29–0.33)
Alpha	1.62 (1.52–1.71)	–	1.25 (1.18–1.31)	1.43 (1.36–1.49)	0.49 (0.46–0.53)
Delta	1.30 (1.23–1.37)	0.80 (0.76–0.84)	–	1.15 (1.11–1.19)	0.40 (0.37–0.42)
Omicron 21K	1.13 (1.08–1.19)	0.70 (0.67–0.73)	0.87 (0.84–0.9)	–	0.35 (0.33–0.37)
Omicron 23A	3.27 (3.06–3.49)	2.02 (1.89–2.16)	2.52 (2.37–2.67)	2.89 (2.73–3.05)	–

Odds ratio comparison of variants in the bolded Y-column in comparison to other variants. All calculations demonstrate statistical significance with *P* value <0.001. Confidence intervals demonstrated in parentheses.

## DISCUSSION

The data we present demonstrate that individuals testing positive for COVID-19 are over twice as likely to develop vestibular symptoms compared to the general population. Although our data estimates the overall prevalence of vestibular disorder amongst these patients at between 1% and 3%, other studies have estimated the self-reported numbers to reach as high as over 40% (7,14,15). One must also consider individuals whose vestibular symptoms remain undiagnosed, those who did not seek medical attention for their vestibular symptoms during our observation window, and those who were never tested and diagnosed with COVID-19.

Vestibular neuritis as an inflammatory process of the vestibular nerve due to acute viral infection or secondary to reactivation of latent neurotropic viruses (eg, herpes simplex virus type 1 or varicella zoster virus) are the second most common cause of vestibular dysfunction, after benign paroxysmal positioning vertigo, with an estimated incidence of 3.5 in 100,000 (16–18). Despite this, our understanding of its pathophysiology remains limited and treatment is mostly centered around symptom management and long-term therapy. Within the context of the COVID-19 pandemic, nearly 700 million individuals were infected with the virus globally with around 100 million of those individuals within the United States at the time of writing. This constitutes over 3 million people with vestibular disorder likely related to COVID-19, with at least 500,000 of those individuals residing in the United States, thus significantly increasing the incidence of vestibular dysfunction secondary to viral etiologies.

A prior study by Almishaal of 939 patients showed a statistically significant increase in subjective, self-reported incidence of audiovestibular symptoms in patients with COVID-19, but did not demonstrate significant differences between viral variants (15). However, it is difficult to assess the clinical significance of subjective, self-reported findings. We aimed to better understand the significance of vestibular symptoms by assessing patients whose symptom severity required medical evaluation. Based on our data, different variants of COVID-19 have differing impacts on the development of vestibular symptoms, with individuals affected by the omicron 23A variant being at highest risk of developing vestibular symptoms, while individuals affected by the initial untyped variant demonstrated a lower risk. It is also important to note that patients with COVID-19-related vestibular symptoms have also demonstrated a different clinical course than the general population, compensating slower compared to the general population (19). Recognizing this phenomenon and intervariant risks may raise clinicians’ suspicions of COVID-19-related vestibular disorder and better guide patient education and treatment options.

As a retrospective cross-sectional study, the biggest limitation of this study is determining the long-term implications and sequelae of vestibular disorder in patients testing positive for COVID-19. Prior studies have suggested that dizziness was one of the most common symptoms lasting greater than 4 weeks in children (20). However, the long-term manifestation and impact of vestibular dysfunction amongst adults is not well-documented in the current literature and would facilitate physicians in understanding the clinical course of COVID-19-associated vestibular disorders and in providing appropriate patient counseling and therapies. Additionally, the diagnosis code queried for “dizziness and giddiness” is general and not specific to otolaryngologists, which may introduce differences in interpretation or misdiagnoses spanning various medical specialties. Similarly, due to the nonspecific nature of the diagnosis code, inferences regarding the vestibular dysfunction such as symptomatology, duration, or severity cannot be deduced. A final limitation is that N3C is sourced from many institutions with multiple source data models that have all been harmonized into the observational medical outcomes partnership common data model. This harmonization process can potentially introduce errors in the data.

## CONCLUSION

Individuals afflicted with COVID-19 are more than twice as likely to develop symptoms of vestibular dysfunction compared to the general population, and the risk of developing vestibular symptoms can vary widely depending upon the variant an individual is impacted by. Continuing to follow trends of how the disease affects patients, further research on the mechanisms causing vestibular symptoms, as well as a better understanding of the long-term impacts these symptoms have on patients will provide a more robust framework for physicians to manage COVID-19-associated vestibular disorder.

## ACKNOWLEDGMENTS

N3C Attribution

The analyses described in this publication were conducted with data or tools accessed through the NCATS N3C Data Enclave https://covid.cd2h.org and N3C Attribution & Publication Policy v 1.2-2020-08-25b supported by NCATS U24 TR002306, Axle Informatics Subcontract: NCATS-P00438-B. This research was possible because of the patients whose information is included within the data and the organizations (https://ncats.nih.gov/n3c/resources/data-contribution/data-transfer-agreement-signatories) and scientists who have contributed to the on-going development of this community resource (https://doi.org/10.1093/jamia/ocaa196).

Disclaimer The N3C Publication Committee confirmed that this manuscript msid:1739.077 is in accordance with N3C data use and attribution policies; however, this content is solely the responsibility of the authors and does not necessarily represent the official views of the National Institutes of Health or the N3C program.

IRB

The N3C data transfer to NCATS is performed under a Johns Hopkins University Reliance Protocol # IRB00249128 or individual site agreements with NIH. The N3C Data Enclave is managed under the authority of the NIH; information can be found at https://ncats.nih.gov/n3c/resources.

Individual Acknowledgements for Core Contributors

We gratefully acknowledge the following core contributors to N3C:

Adam B. Wilcox, Adam M. Lee, Alexis Graves, Alfred (Jerrod) Anzalone, Amin Manna, Amit Saha, Amy Olex, Andrea Zhou, Andrew E. Williams, Andrew Southerland, Andrew T. Girvin, Anita Walden, Anjali A. Sharathkumar, Benjamin Amor, Benjamin Bates, Brian Hendricks, Brijesh Patel, Caleb Alexander, Carolyn Bramante, Cavin Ward-Caviness, Charisse Madlock-Brown, Christine Suver, Christopher Chute, Christopher Dillon, Chunlei Wu, Clare Schmitt, Cliff Takemoto, Dan Housman, Davera Gabriel, David A. Eichmann, Diego Mazzotti, Don Brown, Eilis Boudreau, Elaine Hill, Elizabeth Zampino, Emily Carlson Marti, Emily R. Pfaff, Evan French, Farrukh M Koraishy, Federico Mariona, Fred Prior, George Sokos, Greg Martin, Harold Lehmann, Heidi Spratt, Hemalkumar Mehta, Hongfang Liu, Hythem Sidky, J.W. Awori Hayanga, Jami Pincavitch, Jaylyn Clark, Jeremy Richard Harper, Jessica Islam, Jin Ge, Joel Gagnier, Joel H. Saltz, Joel Saltz, Johanna Loomba, John Buse, Jomol Mathew, Joni L. Rutter, Julie A. McMurry, Justin Guinney, Justin Starren, Karen Crowley, Katie Rebecca Bradwell, Kellie M. Walters, Ken Wilkins, Kenneth R. Gersing, Kenrick Dwain Cato, Kimberly Murray, Kristin Kostka, Lavance Northington, Lee Allan Pyles, Leonie Misquitta, Lesley Cottrell, Lili Portilla, Mariam Deacy, Mark M. Bissell, Marshall Clark, Mary Emmett, Mary Morrison Saltz, Matvey B. Palchuk, Melissa A. Haendel, Meredith Adams, Meredith Temple-O’Connor, Michael G. Kurilla, Michele Morris, Nabeel Qureshi, Nasia Safdar, Nicole Garbarini, Noha Sharafeldin, Ofer Sadan, Patricia A. Francis, Penny Wung Burgoon, Peter Robinson, Philip R. O. Payne, Rafael Fuentes, Randeep Jawa, Rebecca Erwin-Cohen, Rena Patel, Richard A. Moffitt, Richard L. Zhu, Rishi Kamaleswaran, Robert Hurley, Robert T. Miller, Saiju Pyarajan, Sam G. Michael, Samuel Bozzette, Sandeep Mallipattu, Satyanarayana Vedula, Scott Chapman, Shawn T. O’Neil, Soko Setoguchi, Stephanie S. Hong, Steve Johnson, Tellen D. Bennett, Tiffany Callahan, Umit Topaloglu, Usman Sheikh, Valery Gordon, Vignesh Subbian, Warren A. Kibbe, Wenndy Hernandez, Will Beasley, Will Cooper, William Hillegass, Xiaohan Tanner Zhang.

Details of contributions are available at covid.cd2h.org/core contributors.

Data Partners with Released Data

The following institutions whose data is released or pending:

Available: Advocate Health Care Network—UL1TR002389: The Institute for Translational Medicine (ITM); Aurora Health Care Inc—UL1TR002373: Wisconsin Network For Health Research; Boston University Medical Campus—UL1TR001430: Boston University Clinical and Translational Science Institute; Brown University—U54GM115677: Advance Clinical Translational Research (Advance-CTR); Carilion Clinic—UL1TR003015: iTHRIV Integrated Translational Health Research Institute of Virginia; Case Western Reserve University—UL1TR002548: The Clinical & Translational Science Collaborative of Cleveland (CTSC); Charleston Area Medical Center—U54GM104942: West Virginia Clinical and Translational Science Institute (WVCTSI); Children’s Hospital Colorado—UL1TR002535: Colorado Clinical and Translational Sciences Institute; Columbia University Irving Medical Center—UL1TR001873: Irving Institute for Clinical and Translational Research; Dartmouth College—None (Voluntary) Duke University—UL1TR002553: Duke Clinical and Translational Science Institute; George Washington Children’s Research Institute—UL1TR001876: Clinical and Translational Science Institute at Children’s National (CTSA-CN); George Washington University—UL1TR001876: Clinical and Translational Science Institute at Children’s National (CTSA-CN); Harvard Medical School—UL1TR002541: Harvard Catalyst; Indiana University School of Medicine—UL1TR002529: Indiana Clinical and Translational Science Institute; Johns Hopkins University—UL1TR003098: Johns Hopkins Institute for Clinical and Translational Research; Louisiana Public Health Institute—None (Voluntary); Loyola Medicine—Loyola University Medical Center; Loyola University Medical Center—UL1TR002389: The Institute for Translational Medicine (ITM); Maine Medical Center—U54GM115516: Northern New England Clinical & Translational Research (NNE-CTR) Network; Mary Hitchcock Memorial Hospital & Dartmouth Hitchcock Clinic—None (Voluntary); Massachusetts General Brigham—UL1TR002541: Harvard Catalyst; Mayo Clinic Rochester—UL1TR002377: Mayo Clinic Center for Clinical and Translational Science (CCaTS); Medical University of South Carolina—UL1TR001450: South Carolina Clinical & Translational Research Institute (SCTR); MITRE Corporation—None (Voluntary); Montefiore Medical Center—UL1TR002556: Institute for Clinical and Translational Research at Einstein and Montefiore; Nemours—U54GM104941: Delaware CTR ACCEL Program; NorthShore University HealthSystem—UL1TR002389: The Institute for Translational Medicine (ITM); Northwestern University at Chicago—UL1TR001422: Northwestern University Clinical and Translational Science Institute (NUCATS); OCHIN—INV-018455: Bill and Melinda Gates Foundation grant to Sage Bionetworks; Oregon Health & Science University—UL1TR002369: Oregon Clinical and Translational Research Institute; Penn State Health Milton S. Hershey Medical Center—UL1TR002014: Penn State Clinical and Translational Science Institute; Rush University Medical Center—UL1TR002389: The Institute for Translational Medicine (ITM); Rutgers, The State University of New Jersey—UL1TR003017: New Jersey Alliance for Clinical and Translational Science; Stony Brook University—U24TR002306; The Alliance at the University of Puerto Rico, Medical Sciences Campus—U54GM133807: Hispanic Alliance for Clinical and Translational Research (The Alliance); The Ohio State University—UL1TR002733: Center for Clinical and Translational Science; The State University of New York at Buffalo—UL1TR001412: Clinical and Translational Science Institute; The University of Chicago—UL1TR002389: The Institute for Translational Medicine (ITM); The University of Iowa—UL1TR002537: Institute for Clinical and Translational Science; The University of Miami Leonard M. Miller School of Medicine—UL1TR002736: University of Miami Clinical and Translational Science Institute; The University of Michigan at Ann Arbor—UL1TR002240: Michigan Institute for Clinical and Health Research; The University of Texas Health Science Center at Houston—UL1TR003167: Center for Clinical and Translational Sciences (CCTS); The University of Texas Medical Branch at Galveston—UL1TR001439: The Institute for Translational Sciences; The University of Utah—UL1TR002538: Uhealth Center for Clinical and Translational Science; Tufts Medical Center—UL1TR002544: Tufts Clinical and Translational Science Institute; Tulane University—UL1TR003096: Center for Clinical and Translational Science; The Queens Medical Center—None (Voluntary); University Medical Center New Orleans—U54GM104940: Louisiana Clinical and Translational Science (LA CaTS) Center; University of Alabama at Birmingham—UL1TR003096: Center for Clinical and Translational Science; University of Arkansas for Medical Sciences—UL1TR003107: UAMS Translational Research Institute; University of Cincinnati—UL1TR001425: Center for Clinical and Translational Science and Training; University of Colorado Denver, Anschutz Medical Campus—UL1TR002535: Colorado Clinical and Translational Sciences Institute; University of Illinois at Chicago—UL1TR002003: UIC Center for Clinical and Translational Science; University of Kansas Medical Center—UL1TR002366: Frontiers: University of Kansas Clinical and Translational Science Institute; University of Kentucky—UL1TR001998: UK Center for Clinical and Translational Science; University of Massachusetts Medical School Worcester—UL1TR001453: The UMass Center for Clinical and Translational Science (UMCCTS); University Medical Center of Southern Nevada—None (Voluntary); University of Minnesota—UL1TR002494: Clinical and Translational Science Institute; University of Mississippi Medical Center—U54GM115428: Mississippi Center for Clinical and Translational Research (CCTR); University of Nebraska Medical Center—U54GM115458: Great Plains IDeA-Clinical & Translational Research; University of North Carolina at Chapel Hill—UL1TR002489: North Carolina Translational and Clinical Science Institute; University of Oklahoma Health Sciences Center—U54GM104938: Oklahoma Clinical and Translational Science Institute (OCTSI); University of Pittsburgh—UL1TR001857: The Clinical and Translational Science Institute (CTSI); University of Pennsylvania—UL1TR001878: Institute for Translational Medicine and Therapeutics; University of Rochester—UL1TR002001: UR Clinical & Translational Science Institute; University of Southern California—UL1TR001855: The Southern California Clinical and Translational Science Institute (SC CTSI); University of Vermont—U54GM115516: Northern New England Clinical & Translational Research (NNE-CTR) Network Health Research Institute of Virginia; University of Washington—UL1TR002319: Institute of Translational Health Sciences; University of Wisconsin-Madison—UL1TR002373: UW Institute for Clinical and Translational Research; Vanderbilt University Medical Center—UL1TR002243: Vanderbilt Institute for Clinical and Translational Research; Virginia Commonwealth University—UL1TR002649: C. Kenneth and Dianne Wright Center for Clinical and Translational Research; Wake Forest University Health Sciences—UL1TR001420: Wake Forest Clinical and Translational Science Institute; Washington University in St. Louis—UL1TR002345: Institute of Clinical and Translational Sciences; Weill Medical College of Cornell University—UL1TR002384: Weill Cornell Medicine Clinical and Translational Science Center; West Virginia University—U54GM104942: West Virginia Clinical and Translational Science Institute (WVCTSI) Submitted: Icahn School of Medicine at Mount Sinai—UL1TR001433: ConduITS Institute for Translational Sciences; The University of Texas Health Science Center at Tyler—UL1TR003167: Center for Clinical and Translational Sciences (CCTS); University of California, Davis—UL1TR001860: UCDavis Health Clinical and Translational Science Center; University of California, Irvine—UL1TR001414: The UC Irvine Institute for Clinical and Translational Science (ICTS); University of California, Los Angeles—UL1TR001881: UCLA Clinical Translational Science Institute; University of California, San Diego—UL1TR001442: Altman Clinical and Translational Research Institute; University of California, San Francisco—UL1TR001872: UCSF Clinical and Translational Science Institute Pending: Arkansas Children’s Hospital—UL1TR003107: UAMS Translational Research Institute; Baylor College of Medicine—None (Voluntary); Children’s Hospital of Philadelphia—UL1TR001878: Institute for Translational Medicine and Therapeutics; Cincinnati Children’s Hospital Medical Center—UL1TR001425: Center for Clinical and Translational Science and Training; Emory University—UL1TR002378: Georgia Clinical and Translational Science Alliance; HonorHealth—None (Voluntary); Loyola University Chicago—UL1TR002389: The Institute for Translational Medicine (ITM); Medical College of Wisconsin—UL1TR001436: Clinical and Translational Science Institute of Southeast Wisconsin; MedStar Health Research Institute—None (Voluntary); Georgetown University—UL1TR001409: The Georgetown-Howard Universities Center for Clinical and Translational Science (GHUCCTS); MetroHealth—None (Voluntary); Montana State University—U54GM115371: American Indian/Alaska Native CTR; NYU Langone Medical Center—UL1TR001445: Langone Health’s Clinical and Translational Science Institute; Ochsner Medical Center—U54GM104940: Louisiana Clinical and Translational Science (LA CaTS) Center; Regenstrief Institute—UL1TR002529: Indiana Clinical and Translational Science Institute; Sanford Research—None (Voluntary); Stanford University—UL1TR003142: Spectrum: The Stanford Center for Clinical and Translational Research and Education; The Rockefeller University—UL1TR001866: Center for Clinical and Translational Science; The Scripps Research Institute—UL1TR002550: Scripps Research Translational Institute; University of Florida—UL1TR001427: UF Clinical and Translational Science Institute; University of New Mexico Health Sciences Center—UL1TR001449: University of New Mexico Clinical and Translational Science Center; University of Texas Health Science Center at San Antonio—UL1TR002645: Institute for Integration of Medicine and Science; Yale New Haven Hospital—UL1TR001863: Yale Center for Clinical Investigation.

## FUNDING SOURCES

None declared.

## CONFLICT OF INTEREST STATEMENT

None declared.
